# A Comprehensive Numerical Approach for Analyzing the Residual Stresses in AISI 301LN Stainless Steel Induced by Shot Peening

**DOI:** 10.3390/ma12203338

**Published:** 2019-10-13

**Authors:** Fan Zhou, Wenchun Jiang, Yang Du, Chengran Xiao

**Affiliations:** 1 College of New Energy, China University of Petroleum (East China), Qingdao 266580, China; 2 Department of Safety Science and Engineering, China University of Petroleum (East China), Qingdao 266580, China

**Keywords:** shot peening, constitutive model, residual stress, martensitic transformation, numerical approach

## Abstract

Shot peening is one of the most famous mechanical surface treatments to improve fatigue performance of metallic components, which is attributed to high amplitude compressive residual stresses. A numerical approach is developed to analyze the residual stresses in 301LN metastable austenitic stainless steel by shot peening. The material behavior is described by a proposed constitutive model in which strain-induced martensitic transformation, isotropic hardening and kinematic hardening effects are taken into account properly. Both single shot and random multiple shots peening were simulated and analyzed. A numerical method is presented with the Python programming language to make the multiple shots follow a random probability distribution. Results demonstrate that the simulated equivalent plastic strains and martensitic volume fractions agree well with the experimental ones, which verify the validity of the constitutive model. Besides, the numerical method is effective at achieving a realistic surface coverage. The maximum compressive residual stress by the Johnson–Cook model is 12% higher than that of the proposed model. Additionally, each hardening effect has an effect on the simulated residual stress. The developed numerical approach can provide a feasible simulation of the shot-peening process and makes an accurate prediction of the residual stress field in 301LN steel.

## 1. Introduction

Poor surface performance is the main cause of fatigue failure of most metal components. Therefore, improving the surface performance can improve the service life and comprehensive performance of metal components [1]. Shot peening is one of the most famous mechanical surface strengthening treatments due to microstructure improvement and high amplitude compressive residual stresses [2,3]. The strengthening effect depends on multi-factors such as the dynamic mechanical properties of materials and shot-peening process parameters [4]. Therefore, compared with the experimental researches, numerical simulation of the shot-peening process is not only convenient and fast, but also can easily study the individual influence of these factors.

The constitutive model used to describe the target material behavior is an important aspect to carry out reliable numerical simulations of the shot-peening process. For metastable austenitic stainless steels, the severe plastic deformation on the surface by shot peening will cause the strain-induced martensitic transformation [5]. On the one hand, the process of the martensitic transformation is accompanied by an increase in volume, which is called the transformation-induced plasticity (TRIP) phenomenon [6,7]. Two mechanisms are commonly proposed to explain the phenomenon: Greenwood–Johnson [8] and Magee [9]. On the other hand, the combined hardening effects on the surface of material during shot-peening process should be taken into consideration in the constitutive model. Firstly, high-speed impact will cause strain hardening and strain rate hardening on the surface layer, which can be described by isotropic hardening law. The widely used isotropic constitutive models are Johnson–Cook [10] and Cowper–Symonds [11]. Secondly, as the shot-peening process involves the action of repeated loads, the cyclic response of the target material should be assessed. Kinematic hardening law which assumes the yield surface is displaced from its original position in the principal stress space should be applied. The representative models are Armstrong–Frederick [12] and Chaboche [13]. Thirdly, as the yield strength of martensite is usually higher than that of the austensite [14], the strengthening effect on the yield surface due to strain-induced martensitic transformation should be included into the constitutive model.

So far, there are two types of constitutive models developed for metastable austenitic steels: micromechanics-based model and phenomenological macroscopic model. The micromechanics-based model adopts independent constitutive equations for each phase and describes the change of phase volume fraction by an evolution law; the effective behavior of the multiphase material is calculated by homogenization [14,15,16]. In phenomenological macroscopic model, the constitutive equations are established directly on the macroscopic level instead of being given for individual phases. Beese and Mohr [17] have developed an isothermal rate-independent phenomenological macroscopic finite strain plasticity model to exhibit strain-induced austenite-to-martensite transformation. The macroscopic strain hardening is composed of nonlinear kinematic hardening as well as isotropic hardening. Santacreu [18] has proposed a stress-state-independent transformation kinetics model, which introduced the correlation between martensite saturation and stress triaxiality. Sanjurjo [19] has analyzed the influence of different material constitutive models on the numerical simulation of a shot-peening process. It has been found that results by a nonlinear kinematic-isotopic model which considered both effects of isotropic hardening and kinematic hardening were the best compared with other traditional models. Recently, Guiheux [20] has proposed a semi-phenomenological model coupling the martensitic phase transformation and the elastoplastic behavior of austenite and martensite, and studied a single spherical normal shot on strain-induced martensitic transformation in 301LN steel. In this work, the phase transformation, the coupling between plasticity and transformation-induced plasticity, and the mixed hardening effects induced by shot peening will increase the complexity of constructing the constitutive model of the 301LN steel. Although the significance of the target material behavior in the shot-peening simulations has been highlighted, few researchers have taken all these effects into account in the constitutive model.

In addition to the constitutive models, another important aspect in the simulation of a shot-peening process is the finite element model. With the development of finite element method and computational power, six type models have been proposed and widely applied in the simulation of the shot-peening process: 2D axisymmetric model [21], periodic symmetry model with a square contact surface [22], 3D model with three symmetry surfaces [23], 3D model with two symmetry model [24], 3D model with one symmetry surface [25] and 3D model without symmetry boundary condition [26]. In all the above models, the locations of the shots have been pre-defined, which are far from the real shot-peening process. In order to develop a model that can better simulate the shot-peening process, Miao [27] has developed a 3D random finite model with a Matlab program combined with ANSYS APDL; Gangaraj [28] has constructed a 3D random model using a Matlab program and ABAQUS Explicit. Results showed that the random models were superior to the previous models in the simulation of actual surface coverage, surface roughness and residual stress field. However, in these recently developed random models, it is equiprobable for all the entering shots to hit any position on the target surface. Actually, due to the factors such as the nozzle size, touching or impacting between entering shots and rebound shots, it is impossible for the entering shots to hit any position on the target surface with equal probability. Therefore, it is necessary to make the impact dimples follow a random probability distribution.

The objective of the present study is to develop a numerical approach to analyze the shot-peening process and obtain an accurate prediction of the residual stress field in metastable austenitic stainless steel 301LN. The material behavior is described by a proposed constitutive model in which the strain-induced martensitic transformation, isotropic hardening and kinematic hardening effects are taken into account properly, and implemented into ABAQUS by developing a user subroutine. Both single shot and random multiple shots peening were simulated and analyzed. The random multiple shots model with the dimples following a random probability distribution was generated with the Python programming language. The simulated equivalent plastic strains and martensitic volume fractions by a single shot were compared with the experimental results in published literature. Additionally, the relationship between the shot velocities and the dimple diameters was analyzed. Furthermore, the surface coverage, martensitic transformation and residual stresses induced by multiple shots peening were obtained and discussed. In addition, the residual stress field obtained with the proposed constitutive model was compared with that by other constitutive models.

## 2. Proposed Constitutive Model

A constitutive model is proposed based on the previous works [17,18]. In the following, the constitutive equations which include the mechanical behavior law, yield surface, kinematic hardening law, isotropic hardening law and martensitic transformation kinetics law, are outlined.

### 2.1. Mechanical Behavior Law

The total strain increment tensor dε of the 301LN steel which is impacted by shot peening is decomposed into four parts:

(1) $$d\varepsilon =d\varepsilon^{e}+d\varepsilon^{p}+d\varepsilon^{tr}+d\varepsilon^{tp},$$

where $d\varepsilon^{e}$, $d\varepsilon^{p}$ denote respectively the elastic strain increment tensor and plastic strain increment tensor; $d\varepsilon^{tr}$, $d\varepsilon^{tp}$ are respectively the volume strain increment tensor between martensitic and austenite phases due to density difference and the plasticity strain increment tensor induced by phase transformation.

It is assumed that the stress–strain relationship of elasticity conforms to isotropic Hooke’s law, and the calculation formula is as follows:

(2) $$d\varepsilon^{e}=\frac{1+v}{E}d\sigma -\frac{v}{E}d\sigma_{k}\delta ,$$

where E is the elasticity modulus, which is a homogenization of elasticity modulus according to the volume fraction of martensitic and austenite phases; *v* is the Poisson’s ratio; dσ is the total stress increment tensor; $\sigma_{k}$ is the mean stress increment tensor; δ is the Kronecker symbol tensor.

Plastic strain increment is calculated using the plastic flow criterion:

(3) $$d\varepsilon^{p}=\frac{3S}{2\sigma_{e}}\sqrt{\frac{3}{2}d\varepsilon^{p}:d\varepsilon^{p}},$$

where S is the deviatoric stress tensor; $\sigma_{e}$ denotes the equivalent stress.

The strain due to phase volume change, $d\varepsilon^{tr}$, is defined as [29]:

(4) $$d\varepsilon^{tr}=\left(\sum 2^{w}\beta_{x}dX_{x}\right)\delta ,$$

where $\beta_{x}$ denotes the phase transformation parameter; $dX_{x}$ is the volume fraction increment of each phase; *x* = 1, 2 denotes the martensitic and austenite phases respectively; *w* = 2 is the number of the phases.

According to the Greenwood–Johnson model, the plasticity strain increment tensor due to phase transformation $d\varepsilon^{tp}$ is defined as:

(5) $$d\varepsilon^{tp}=3M\left(1-f\right)dfS,$$

where *M* is the parameter of transformation plasticity; *f* is the martensite volume fraction; df is the martensite volume fraction increment.

### 2.2. Yield Surface

The initial yield function *F* of the 301LN steel is given by:

(6) $$F=\sigma_{e}-K=\sqrt{\frac{3}{2}\left(S-\alpha \right):\left(S-\alpha \right)}-K,$$

where α is the back stress tensor; *K* denotes the yield stress, which is defined in the following.

### 2.3. Kinematic Hardening Law

The nonlinear kinematic hardening rule proposed by Armstrong–Frederick [12] is adopted. The evolution of the back stress is written as:

(7) $$d\alpha =\frac{2}{3}r\theta d\varepsilon^{p}-\theta \alpha dp,$$

where dα denotes the increment of back stress tensor; r,θ are material parameters; dp defines the increment of equivalent plastic strain.

### 2.4. Isotropic Hardening Law

In addition to the kinematic hardening law, an isotropic hardening law is used to describe the evolution of the yield stress during shot-peening process. The widely used isotropic hardening model is the Johnson–Cook model, which takes the strain hardening and strain rate hardening into account:

(8) $$\sigma =\left(A+Bp^{n}\right)\left[1+C\ln\left(\dot{p}/\dot{\varepsilon_{0}}\right)\right],$$

where σ denotes the yield stress in this model; *A*, *B*, *C* and *n* are the model parameters; $\dot{p}$ is the equivalent plastic strain rate; $\dot{\varepsilon_{0}}$ is the reference plastic strain rate.

In this work, yield stress *K* depends on the equivalent plastic strain, the equivalent plastic strain rate and the martensitic volume fraction. Based on the Johnson–Cook model, an isotropic hardening law is proposed as follows:

(9) $$K=\left(k_{\varepsilon }+k_{f}\right)\left[1+C\ln\left(\dot{p}/\dot{\varepsilon_{0}}\right)\right],$$

(10) $$k_{\varepsilon }=\sigma_{0}+h_{\varepsilon }\left[1-\exp\left(-ap\right)\right],$$

(11) $$k_{f}=h_{f}f^{n},$$

where $k_{\varepsilon }$ denotes the strain hardening effect which is expressed as an exponential function of the equivalent plastic strain; $k_{f}$ denotes the martensite transformation hardening effect which is an function of the martensite volume fraction; $\sigma_{0}$ is the initial yield stress of 301LN steel; $h_{\varepsilon }$, *a* and *n* are the material parameters; $h_{f}$ denotes the martensitic transformation hardening modulus.

### 2.5. Martensitic Transformation Kinetics Law

According to the transformation kinetics law by Santacreu [18], the incremental equation of the martensitic volume fraction, *df*, is given as:

(12) $$df=\left(f_{max}-f\right)mD{\left(Dp\right)}^{m-1}dp,$$

(13) $$D=D_{0}+D_{1}\varphi ,$$

where $f_{max}$ denotes the maximum of martensitic volume in the 301LN steel during shot peening process; *D*, $D_{0}$, $D_{1}$ and m are the material parameters; φ denotes the stress triaxiality.

### 2.6. Identification of the Constitutive Model Parameters

The data from the published literature [17] which correspond to the parameters of the proposed constitutive model in this work are listed in Table 1. These parameters associated with the isotropic hardening, kinematic hardening and transformation kinetics have been calibrated respectively based on the experiments. The parameters of the proposed model in this work are identified though numerical optimizations, which are carried out on a single cubic element. The parameters in Table 1 are determined as the initial parameters. Other parameters which are included in the proposed model but not listed in Table 1 are estimated. The constitutive model has been implemented in the finite element software ABAQUS by developing a user subroutine. Because we are going to compare the simulated results of the shot peening with that in the literature [20], the material mechanical behaviors from tensile tests in Reference [20] are taken as the targets of the optimizations.

**Figure 1:**
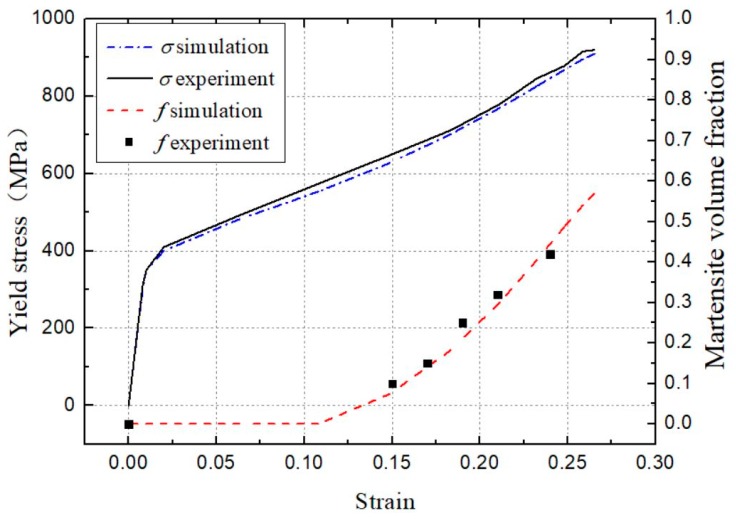
Evolution of experimental and simulated yield stress and martensitic volume fraction.

Table 2 shows the final values of the proposed model parameters. Figure 1 presents the simulated behavior curves. It can be seen that the yield stress and the martensitic volume fraction agrees well with the experimental results from the literature [20]. The martensitic transformation starts at the strain of about 12% and increases to 0.6 of the volume fraction at the strain of 0.25. As the yield strength of martensite is higher than that of the austenite, the yield stress starts form 350 MPa and increases up to 900 MPa with the martensitic volume fraction.

## 3. Finite Element Modelling

As the shot peening is a high-speed dynamic process, an explicit solver in the ABAQUS is adopted in this work. Both axisymmetric single shot and random multiple shots models were developed. For comparison, the simulated conditions of the axisymmetric single shot model were according to the experimental ones [20]. The target specimen was modelled as a rectangle with a width of 35 mm and 25 mm in height. The degrees of the bottom edges were restricted. The diameter of shot was set as 10 mm.

To simulate the real shot-peening process, a 3D random multiple shots model was developed with the Python programming language. The initial location of the shot centers was generated using the random function, Random. Uniform ( ). The three dimensional coordinates of the shot center, *r*, *θ*, *z*, were all limited within a space. To avoid the overlap of the created shots, the distance between the centers of any two shots was set to be greater than the shot diameter. Due to factors such as the touching or impacting between entering shots and rebound shots, it was impossible for the entering shots to hit any position on the target surface with equal probability. Therefore, to make the dimples by impacting follow a random probability distribution, the Avrami equation [30] was applied to evaluate the relative position relationship between the two dimples:

(14) P(l)=100%(1−exp(−ε∗l/∅)),

where P(l) denotes the probability that the distance between two dimples is *l*; ∅ is the diameter of the dimple; ε is a constant. The P(l) must not be less than a random value which is produced by the random function, Random. Uniform (0, 1). Otherwise, the shot creation will be rejected and the location of a new shot will be generated until all the criteria are met.

The 3D target specimen was modelled as a cylinder with a radius of 5 mm and 3 mm in height. The treated rectangular area on the target specimen surface was determined as 3 mm × 3 mm. The degrees of freedom at the bottom face were constrained. To get close to the actual shot-peening process, the diameter of the shots was set as 2.0 mm in the multiple shots model. In both models, each shot was modelled as a rigid solid with the density of 7800 kg/m^3^, and defined by a reference point corresponding to its center. In this work, the contacts between shots were ignored for simplicity. Only the contacts between the shots and the target surface were defined with the coefficient of friction of 0.2. The impact angle was 90° in order to maximize the impact energy transferred to the surface.

The single shot model was meshed using quadrilateral elements with reduced integration CAX4R. Infinite elements (CINAX4) were employed to cover the lateral and bottom edges to eliminate the size effect and the reflections of the elastic wave. The 3D multiple shots model was meshed using 8-node continuum elements with reduced integration and hourglass control (C3D8R). Infinite elements (CIN3D8) were employed to cover the lateral faces.

**Figure 2:**
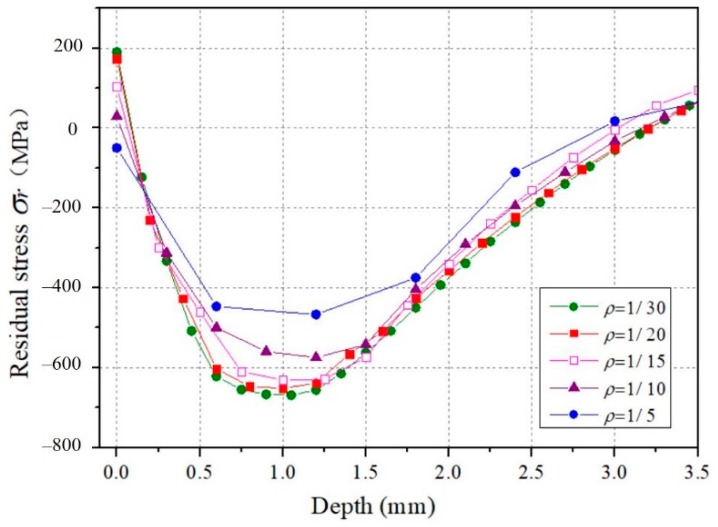
Effect of different values of *ρ* on residual stress *σ_r_* along the depth.

**Figure 3:**
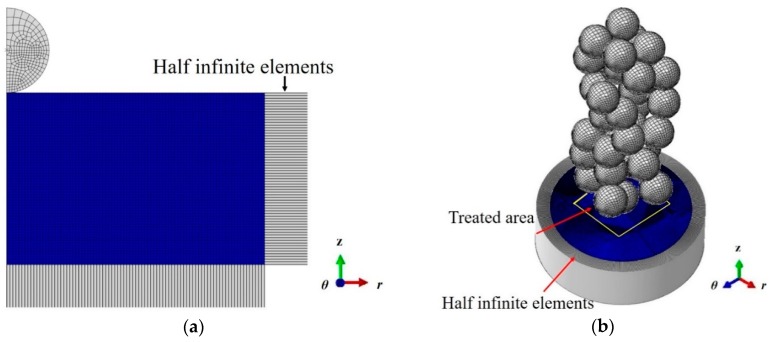
(**a**) Single shot model; (**b**) 3D random multiple shots model.

In Explicit finite element simulation, the minimum element size is of great significance for calculation accuracy and efficiency. A few studies on mesh convergence have been carried out. Frija [31] has conducted a sensitivity study to optimize the dimensions of the element in refined zone by comparison of the analytical solutions of the elastic Hertz contact problem. Klemenz [32] has used the size of elements equal to one-tenth of the dimple diameter in the multiple shots model. Bagherifard [33] has chosen the size of element in the impact zone equal to one–twentieth of the dimple diameter. Obviously, the accuracy of the results mainly depends on the ratio of the element size and the dimple diameter. Define the parameter *ρ* for the ratio of element size to the dimple diameter. A single shot impact was simulated to determine the suitable element size of the target material. Firstly, the diameter of the dimple on the surface was estimated. After that, a mesh convergence study with different values of *ρ* was carried out. The effect of *ρ* on residual stress *σ_r_* along the depth is shown in Figure 2. It represents that the residual stress distributions for *ρ* = 1/20 and *ρ* = 1/30 are almost identical. So it can be inferred that stopping the refinement of the mesh at *ρ* = 1/20 does not result in excessive variation of residual stress. Eventually, a finer mesh was used on the impact zone with a minimum element size of 0.15 mm for the single shot-peening simulation, and 0.03 mm for the random multiple shot-peening simulation. The developed single shot and 3D random multiple shot models are shown in Figure 3a,b respectively. The calculations were running on a computer with 4-core CPU (Central Processing Unit) at 3.4 GHz.

## 4. Results and Discussions

### 4.1. Single Shot-Peening Simulation

**Figure 4:**
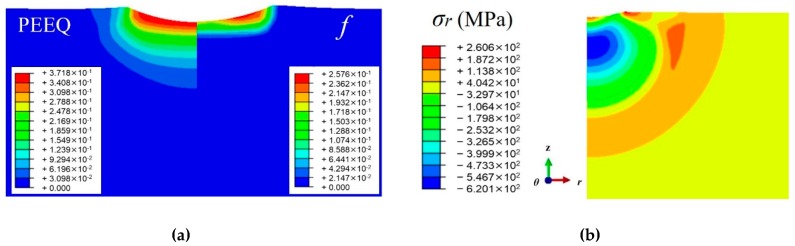
Distributions of (**a**) equivalent plastic strain (PEEQ) and martensitic volume fraction (*f*); (**b**) radial residual stress (*σ_r_*).

Figure 4a,b show the distributions of the simulated equivalent plastic strain (PEEQ), the martensitic volume fraction (*f*) and the radial residual stress *σ_r_* of 301LN steel after being impacted by a single shot of 10 mm in diameter at velocity of 35 m/s. It can be seen that the PEEQ is maximum at the surface and decreases gradually along the depth. However, the martensitic volume fraction appears only in the dimple where the strain is higher than 0.12. Guiheux [20] has investigated a single shot with the same diameter as in this work on the 301LN steel by simulations and experiments. Our results agrees well with Guiheux’s work.

**Figure 5:**
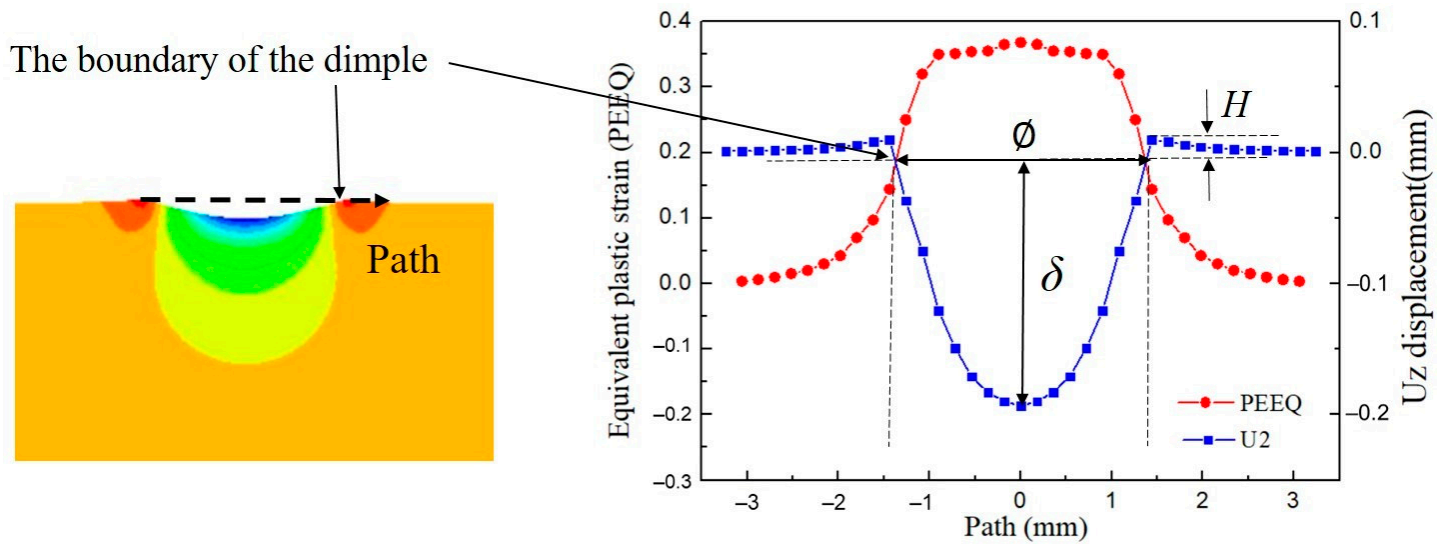
Definitions of the dimensions of the dimple and the height of the pile-up.

As we know, the experimental measurement of the shot velocities has high complexity [34,35]. However, the size of dimple by impact can be easily obtained by measuring. Therefore, it is feasible to relate the shot velocities and the dimple sizes in order to evaluate the shot velocities. The first thing to do is to obtain the dimple diameter. Figure 5 shows the normal displacement (Uz) and the PEEQ along the path which is defined across the center axis of dimple. In this work, the nodes with the Uz be equal to zero on the surface within the pile-up are defined as the boundary of the dimple. Therefore, the dimple diameter Ø, the dimple depth *δ* and the height of the pile-up *H* can be obtained. From Figure 5, the PEEQ at the boundary is 0.19. According to Miao’s definition [27], the points on the surface with the PEEQ larger than 0.19 can be considered as the impacted material. This definition will be applied in the following coverage simulation.

**Figure 6:**
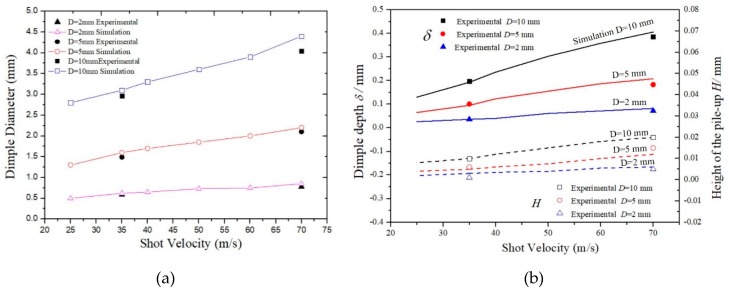
Evolutions of the dimensions of the dimple as a function of the shot velocities: (**a**) the dimple diameter; (**b**) the dimple depth and height of the pile-up.

In single shot-peening simulations, the dimple diameters as a function of shot velocities are obtained. Figure 6a accordingly shows the plots of three different shot diameters: 2, 5, and 10 mm. Experimental results from Reference [20] are also be plotted in the figure. Additionally, the dimple depth *δ* and the height of the pile-up *H* are plotted and compared with the experimental data, as shown in Figure 6b. It can be seen that the simulated results are in good agreement with the experimental results. As expected, an increase in velocity causes an increase in the dimensions of the dimple, and an increase in shot diameter also causes an increase in the dimensions of the dimple. Besides, just by knowing the dimple diameter which are easily obtained experimentally, the shot velocity can be deduced by simply referring to the right shot diameter curve.

### 4.2. Surface Coverage by Random Multiple Shots Simulation

Surface coverage, which indicates the amount of target area treated by shot peening, is an important parameter to evaluate the shot-peening process [28,36]. The relationship between coverage and number of shots (or shot-peening time) is an urgent issue needed to be solved. Most studies have been carried out based on experiments and theoretical models [37,38]. Only few studies have related the coverage to the number of shots by numerical simulation. Recently, Avrami equation has been applied to calculate the number of shots needed to achieve full coverage in simulation [39]. The equation is given by:

(15) $$C\%=100\left(1-\exp\left(-A_{\emptyset }\right)\right),$$

where C% is the theoretical coverage percentage; $A_{\emptyset }$ is the ratio of total dimple area to the target area.

**Figure 7:**
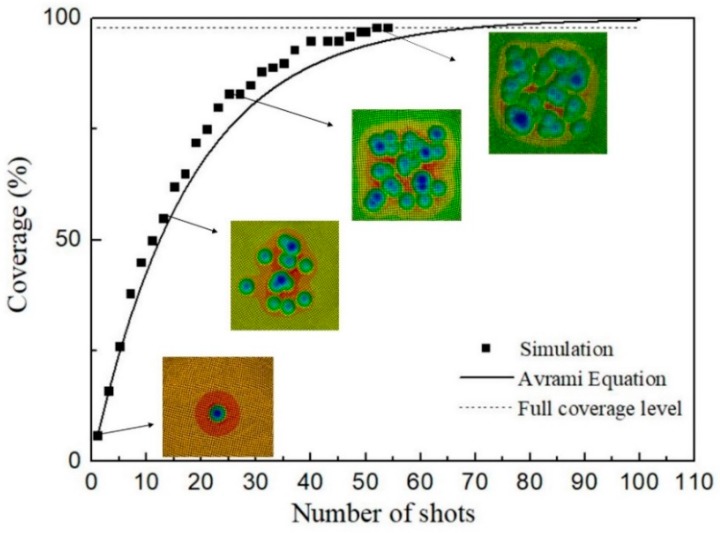
Coverage versus the number of shots: simulation and Avrami equation.

In this work, the coverage is defined as the ratio of the number of nodes with PEEQ larger than that at the boundary of the dimple, to the total number of nodes in the target area according to Miao’s work [27]. For practical purposes, 98% is considered to be full coverage level [39]. Figure 7 shows the variation of the coverage with the number of shots of 2 mm in diameter at velocity of 35 m/s. It can be seen that at first stage the coverage increases quickly with the number of shots increasing. Then with the probability of overlap increasing, the increase rate slows down and the coverage approaches a full level. The development of the coverage by Avrami equation is also superimposed in Figure 7. The simulated results accords basically with the calculated results by Avrami equation within low coverage. However, the results by Avrami equation overestimates the required number of shots needed to achieve the full coverage. The number calculated by Avrami equation is 68. However, the full coverage can be acquired after 55 impacts in the random shot-peening simulation. The reason may be that the size of the treated area in the simulation is not very appropriate for the application of Avrami equation. Other research has reported that the treated area was at least 100 times of the single dimple area [28]. In this work, the area is only about 32 times of the single dimple area. Therefore, random multiple shots model is a reliable method to achieve a realistic surface coverage. Moreover, when applying the Avrami equation to evaluate the number of shots needed to achieve the full coverage, dimension of the treated area needs to be calculated considering the effects of the shot diameter, the shot velocity and the target material.

**Figure 8:**
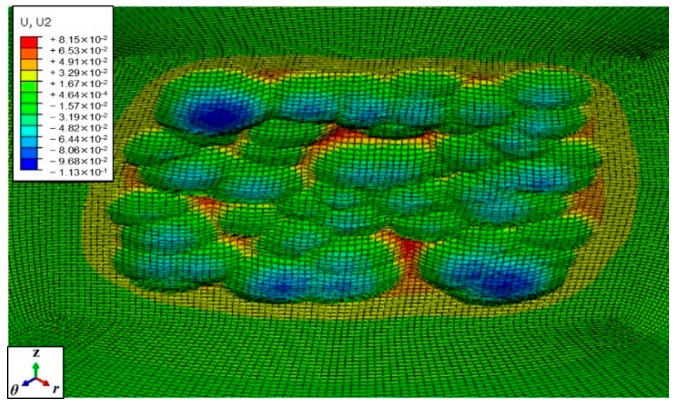
Vertical displacements of the surface nodes after multiple shots peening.

Figure 8 shows the final appearance of the target material after being impacted at full coverage. As a result of the random probability distribution of multiple impacts, the target surface does not show a completely uniform distribution of vertical displacements.

### 4.3. Residual Stress Field by Random Multiple Shots Simulation

**Figure 9:**
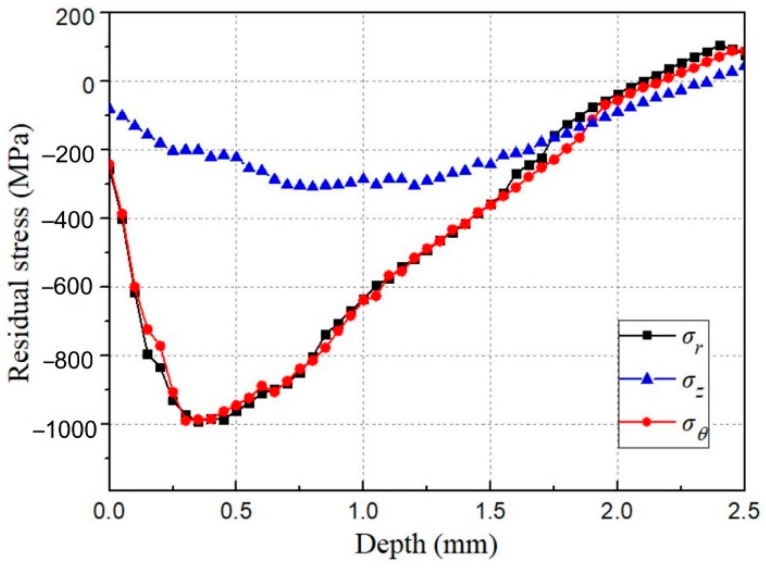
Distributions of the residual stresses along the depth in three directions.

One of the most important results for shot-peening simulation to obtain is the residual stress field, which is a key factor to improve fatigue resistance of metallic components. Figure 9 shows the distributions of residual stresses in directions of *r*, *θ* and *z* respectively after being impacted by 55 shots of 2 mm in diameter at velocity of 35 m/s. It can be seen that the curves of *σ_r_* and *σ_θ_* along the depth are nearly overlap which indicates that the stresses are independent of the measuring direction and therefore the model is reliable. The compressive stress *σ_r_* (*σ_θ_*) is of −240 MPa at the surface and increases until a maximum compressive stress of nearly −1000 MPa at 0.3 mm. It indicates that the material hardening is obvious, especially between 0 and 1mm. The energy provided by shot peening is mostly used in the plastic deformation of deeper layers. The residual stress *σ_z_* is relatively uniform along the depth. The maximum value is −300 MPa, which is much less than the residual stresses in other directions.

**Figure 10:**
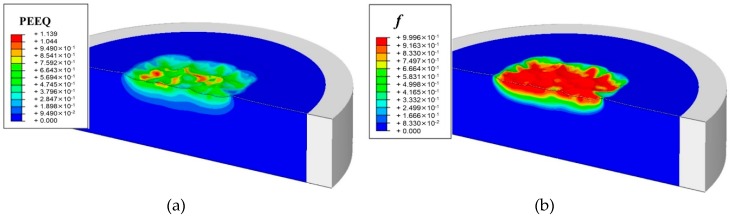
Distributions of (**a**) equivalent plastic strain, PEEQ; (**b**) the martensitic volume fraction, *f*.

**Figure 11:**
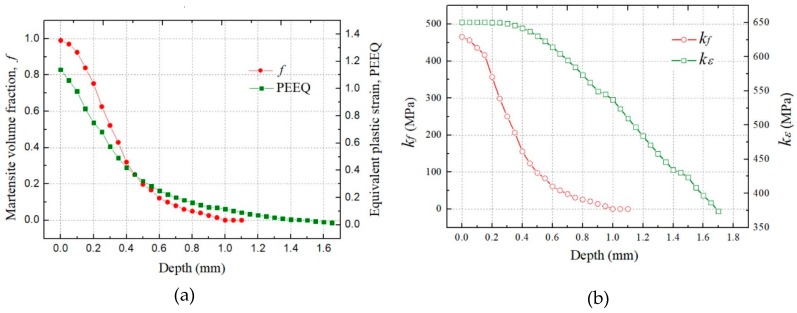
Distributions of variables along the depth by multiple shots peening: (**a**) PEEQ and *f*; (**b**) *k_ε_* and *k_f_*.

Figure 10a,b show the simulated equivalent plastic strain (PEEQ) and the martensitic volume fraction (*f*) of 301LN steel after multiple shots peening. It can be seen that both the PEEQ and *f* are maximum at the surface. Distributions of the PEEQ and *f* along the depth are both plotted in Figure 11a. PEEQ is of 1.139 at surface and decreases until zero at 1.65 mm, while *f* is of 0.99 at surface and decreases until zero at 1.1mm. It means that the austenite phase has been completely transformed into martensitic phase on the surface, while no martensitic transformation occurs under the depth of 1.1 mm.

The maximum compressive residual stress in Figure 9 is much higher than the initial yield stress $\sigma_{0}$, which is attributed to the increase of the yield stress because of hardening effects induced by shot peening. The variables $k_{\varepsilon }$ and $k_{f}$ in the proposed constitutive model (Equations (10) and (11)) which denote the plastic strain hardening effect and the martensite transformation hardening effect have been obtained in the simulations and plotted in Figure 11b respectively. Both variables are maximum at the surface and decrease along the depth. It is interesting to note that the depth of enhancement layer because of martensite phase transition is within 1.1 mm, and the thickness of the work-harden layer is about 1.65 mm. With the depth increases, the effects of martensitic transformation and isotropic hardening on the residual stresses gradually weaken.

**Figure 12:**
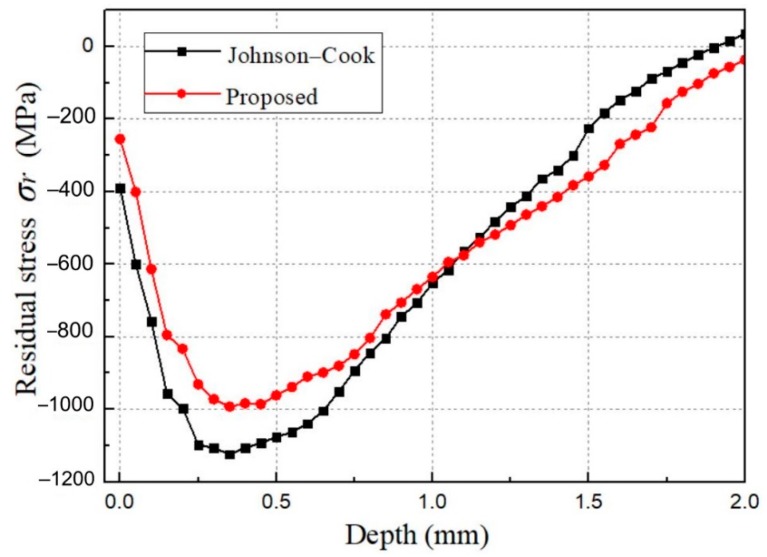
Distributions of the residual stresses along the depth by the Johnson–Cook and the proposed constitutive models.

Distributions of the residual stress *σ_r_* simulated with the Johnson–Cook model and the proposed model are compared, as shown in Figure 12. The Johnson–Cook model describes the evolution of the hardening plastic surface in terms of the plastic strain and the equivalent plastic strain (Equation (8)). The parameters of the Johnson–Cook model as listed in Table 3 are determined by numerical optimization, the goal of which is to fit the curve of yield stress–strain in Figure 1 as well as possible. As Figure 12 shows, the result differences between these models are significant in the surface layer within 1.0 mm. Additionally, the difference is reduced in greater depth where the response of the target material behavior to shot-peening process is weakening. This result corresponds to that obtained in Figure 11. Moreover, it can be seen that the variation trends of the residual stresses and the locations of the maximum values are almost the same. However, the values of maximum compressive residual stress predicted by the constitutive models vary greatly. The value by the Johnson–Cook model is about 12% higher than that by the proposed model. The results are almost consistent with Sanjurjo’s work [19], in which they found that the numerical prediction by the Johnson–Cook model overestimated the residual stress. Results confirm that both the effects of TRIP, isotropic hardening and kinematic hardening have to be properly taken into consideration in the constitutive model of 301LN steel in order to make an accurate prediction of the residual stress field by shot peening.

## 5. Conclusions

(1) A constitutive model considering the effects of strain-induced martensitic transformation, isotropic hardening and kinematic hardening of 301LN steel induced by shot peening is proposed, and implemented into ABAQUS. The validity of the constitutive model was verified by comparison with the experimental models in other published literature.

(2) A numerical method was developed with the Python programming language to make the shots follow a random probability distribution. The Avrami equation was applied to evaluate the relative position relationship between the two impact dimples. The result shows that it is an effective method to achieve a realistic surface coverage by shot peening.

(3) The maximum compressive residual stress predicted by the Johnson–Cook model is 12% higher than that of the proposed model, the value by Johnson–Cook model is 12% higher. Each hardening effect considered in the model has an effect on the residual stress distribution.

Results indicate that the developed numerical approach can provide a feasible simulation of the shot-peening process and make an accurate prediction of the residual stress field in 301LN steel.

## Figures and Tables

**Table 1:** Data from the published research [17].

*r*/MPa	*θ*	*σ*_0_/MPa	*h_ε_*/MPa	*a*	*h_f_*/MPa	*D*_0_	*D*_1_	*f_max_*	*m*
2	767	963	103	5.6	396	6.0	3.0	1.0	1.5

**Table 2:** Parameters of the proposed constitutive model.

*r*/MPa	*θ*	*σ*_0_/MPa	*h_ε_*/MPa	*C*	*a*	*h_f_*/MPa
2.5	300	350	301	0.013	8.37	470
*n*	*D*_0_	*D*_1_	*f_max_*	*m*	*β_x_*	*M*
0.97	2.62	0.6	0.99	2.22	1.0	5.9 × 10^−11^

**Table 3:** Parameters of the Johnson–Cook model.

*A*/MPa	*B*/MPa	*C*	*n*
350	420	0.015	0.5

## Appendix A. Computational Model

The incremental integration of the constitutive model is regarded as a strain-driven process, where the strain tensor increment is given at a certain time and both stresses and state variables (strain, stress, back stress tensor, martensitic volume fraction, equivalent plastic strain and yield surface) should be updated. Therefore, the return mapping algorithm is applied to solve equations. In all the equations below, the subscript *n* + 1 denotes the value of the variables at the time of *n* + 1, while the subscript *n* denotes the value of the variables at the time of *n*.

The total stress tensor is written as:

(A1) $$\sigma_{n+1}^{total}=\sigma_{n+1}^{trial}+\sigma_{n+1}^{tr}+\sigma_{n+1}^{tp},$$

where $\sigma_{n+1}^{trial}$ is the trial stress tensor; $\sigma_{n+1}^{tr}$ is the stress tensor due to volume strain increment; $\sigma_{n+1}^{tp}$ denotes the stress tensor due to the plasticity strain increment induced by phase transformation.

Assume that the strain increment is elastic, and calculate the stress tensor increment according to the generalized Hooke’s law. The trial stress tensor $\sigma_{n+1}^{trial}$ is given by:

(A2) $$\sigma_{n+1}^{trial}=\sigma_{n}+\Delta \sigma_{n+1}=\sigma_{n}+E_{n+1}:\Delta \varepsilon_{n+1},$$

where $\sigma_{n}$ is the stress tensor at the end of time *n*; $\Delta \sigma_{n+1}$ is the increment of the trial stress tensor; $E_{n+1}$ is a homogenization of elasticity modulus according to the volume fraction of martensitic and austenite phases; $\Delta \varepsilon_{n+1}$ is the increment of total strain tensor.

$\sigma_{n+1}^{tr}$ and $\sigma_{n+1}^{tp}$ are respectively given by:

(A3) $$\sigma_{n+1}^{tr}=-E_{n+1}:\Delta \varepsilon_{n+1}^{tr},$$

(A4) $$\sigma_{n+1}^{tp}=-E_{n+1}:\Delta \varepsilon_{n+1}^{tp},$$

With

(A5) $$\Delta \varepsilon_{n+1}^{tr}=\left(\sum_{x}2^{w}\beta_{x}\Delta X_{n+1}^{x}\right)\delta ,$$

(A6) $$\Delta \varepsilon_{n+1}^{tp}=3M\left(1-f_{n+1}\right)\Delta f_{n+1}S_{n+1},$$

where ∑ is the summation notation. $\beta_{x}$ denotes the phase transformation parameter; $\Delta X_{n+1}^{x}$ denotes the volume fraction increment of each phase; *x* = 1, 2 denotes the martensitic and austenite phases respectively; *w* = 2 is the number of the phases.

The volume fraction increment of the martensitic phase is given by:

(A7) $$f_{n+1}=f_{n}+\Delta f_{n+1}=f_{n}+\left(f_{max}-f_{n+1}\right)mD{\left(Dp_{n+1}\right)}^{m-1}\Delta p_{n+1}.$$

The yield function $F_{n+1}$ is defined by:

(A8) $$F_{n+1}=\sigma_{e}-K_{n+1}=\sqrt{\frac{3}{2}\left(S_{n+1}-\alpha_{n+1}\right):\left(S_{n+1}-\alpha_{n+1}\right)}-K_{n+1}.$$

The back stress tensor is defined as:

(A9) $$\alpha_{n+1}=\alpha_{n}+\frac{2}{3}r\theta \Delta \varepsilon_{n+1}^{p}-\theta \alpha_{n+1}\Delta p_{n+1},$$

where $\alpha_{n}$ is the back stress tensor at time *n*; $\Delta \varepsilon_{n+1}^{p}$ is the increment of plastic strain tensor; $\Delta p_{n+1}$ is the equivalent plastic strain increment.

When $F_{n+1}<0$, the new stress is set equal to the trial stress. When $F_{n+1}\geq 0$, the material reaches a yield state. The total strain increment includes both elastic strain increment and plastic strain increment. Therefore, the trial stress is not equal to the true new stress. The trial stress tensor can also be written as:

(A10) $$\sigma_{n+1}^{trial}=\sigma_{n}+\lambda tr\left(\Delta \varepsilon_{n+1}\right)I+2G\Delta \varepsilon_{n+1},$$

where *tr*() is the trace function; I is the unit matrix; λ and *G* are the Lames constants for the material; $\Delta \varepsilon_{n+1}$ denotes the total strain increment tensor. The new stress $\sigma_{n+1}$ is obtained by subtracting the component of plastic strain increment from the total strain increment, as follows:

(A11) $$\sigma_{n+1}=\sigma_{n}+\lambda tr\left(\Delta \varepsilon_{n+1}-\Delta \varepsilon_{n+1}^{p}\right)I+2G\left(\Delta \varepsilon_{n+1}-\Delta \varepsilon_{n+1}^{p}\right).$$

Considering $tr\left(\Delta \varepsilon_{n+1}^{p}\right)=0$, then:

(A12) $$\sigma_{n+1}=\sigma_{n}+\lambda tr\left(\Delta \varepsilon_{n+1}\right)I+2G\Delta \varepsilon_{n+1}-2G\Delta \varepsilon_{n+1}^{p}=\sigma_{n+1}^{trial}-2G\Delta \varepsilon_{n+1}^{p}.$$

When the new stress is expressed in terms of deviatoric stress and mean stress, the Equation (A12) can be rewritten as:

(A13) $$S_{n+1}+\frac{1}{3}tr\left(\sigma_{n+1}\right)I=\sigma_{n+1}^{trial}-2G\Delta \varepsilon_{n+1}^{p}.$$

By sorting out the Equation (A13), we get

(A14) $$S_{n+1}+2G\Delta \varepsilon_{n+1}^{p}=\sigma_{n+1}^{trial}-\frac{1}{3}tr\left(\sigma_{n+1}\right)I=S_{n+1}^{*}.$$

where $S_{n+1}^{*}$ denotes the deviatoric stress tensor of the trial stress $\sigma_{n+1}^{trial}$.

Therefore, the deviatoric stress tensor $S_{n+1}$ is given by:

(A15) $$S_{n+1}=S_{n+1}^{*}-2G\Delta \varepsilon_{n+1}^{p}.$$

Based on the plastic flow law associated with the yield law, it can be drawn that

(A16) $${\dot{\varepsilon }}_{n+1}^{p}=\dot{\lambda }\frac{\partial F_{n+1}}{\partial \sigma_{n+1}}=\dot{\lambda }N_{n+1},$$

where ${\dot{\varepsilon }}_{n+1}^{p}$ denotes the rate of the plastic strain tensor; $\dot{\lambda }$ is a scalar multiplier; $N_{n+1}$ denotes the normal direction outside the yield surface, it can be expressed as:

(A17) $$N_{n+1}=\sqrt{\frac{3}{2}}\frac{S_{n+1}-\alpha_{n+1}}{K_{n+1}}.$$

When the incremental step is small, Equation (A16) can be rewritten as:

(A18) $$\Delta \varepsilon_{n+1}^{p}=\frac{3}{2}\Delta p_{n+1}\frac{S_{n+1}-\alpha_{n+1}}{K_{n+1}}.$$

Using Equations (A9), (A15) and (A18), we obtain:

(A19) $$S_{n+1}-\alpha_{n+1}=\frac{K_{n+1}\left(S_{n+1}^{*}-\frac{\alpha_{n}}{\left(1+\theta \Delta p_{n+1}\right)}\right)}{K_{n+1}+\left(3G+\frac{r\theta }{\left(1+\theta \Delta p_{n+1}\right)}\right)\Delta p_{n+1}}.$$

Substitute Equation (A19) into the yield function $F_{n+1}=0$, we obtain:

(A20) $$\sqrt{\frac{3}{2}\left(S_{n+1}^{*}-\mu \right):\left(S_{n+1}^{*}-\mu \right)}-\left(3G+\varphi \right)\Delta p_{n+1}-K_{n+1}=0,$$

where $\mu =\frac{\alpha_{n}}{\left(1+\theta \Delta p_{n+1}\right)}$; $\varphi =\frac{r\theta }{\left(1+\theta \Delta p_{n+1}\right)}$; $K_{n+1}=\left(\sigma_{0}+h_{\varepsilon }\left[1-\exp\left(-ap_{n+1}\right)\right]+h_{f}f_{n+1}^{n}\right)\left[1+C\ln\left(\frac{\dot{p_{n+1}}}{\dot{\varepsilon_{0}}}\right)\right]$.

The equivalent plastic strain increment $\Delta p_{n+1}$ is obtained by solving Equation (A13) with the Newton iteration method:

(A21) $$\Delta p_{n+1}=\Delta p_{i}=\Delta p_{i-1}-\frac{F_{0}\left(\Delta p_{i-1}\right)}{F_{0}^{\prime }\left(\Delta p_{i-1}\right)},$$

where *i* and *i* − 1 denote the number of iterations; $\Delta p_{i}$ is the equivalent plastic strain increment after the *i* iteration; $\Delta p_{i-1}$ is the equivalent plastic strain increment after the *i* − 1 iteration. The iterative procedure is continued until the criterion for accuracy requirement is met.
